# Multiple Bactericidal Mechanisms of the Zinc Ionophore PBT2

**DOI:** 10.1128/mSphere.00157-20

**Published:** 2020-03-18

**Authors:** Nichaela Harbison-Price, Scott A. Ferguson, Adam Heikal, George Taiaroa, Kiel Hards, Yoshio Nakatani, David Rennison, Margaret A. Brimble, Ibrahim M. El-Deeb, Lisa Bohlmann, Christopher A. McDevitt, Mark von Itzstein, Mark J. Walker, Gregory M. Cook

**Affiliations:** a Department of Microbiology and Immunology, University of Otago, Dunedin, New Zealand; b School of Chemical Sciences, University of Auckland, Auckland, New Zealand; c Institute for Glycomics, Griffith University, Queensland, Australia; d School of Chemistry and Molecular Biosciences, Australian Infectious Diseases Research Centre, The University of Queensland, Queensland, Australia; e Department of Microbiology and Immunology, The Peter Doherty Institute for Infection and Immunity, University of Melbourne, Melbourne, Victoria, Australia; University of Rochester

**Keywords:** antimicrobial resistance, PBT2, zinc, manganese, ionophore, metal ion homeostasis, oxidative stress

## Abstract

Globally, more antimicrobials are used in food-producing animals than in humans, and the extensive use of medically important human antimicrobials poses a significant public health threat in the face of rising antimicrobial resistance (AMR). The development of novel ionophores, a class of antimicrobials used exclusively in animals, holds promise as a strategy to replace or reduce essential human antimicrobials in veterinary practice. PBT2 is a zinc ionophore with recently demonstrated antibacterial activity against several Gram-positive pathogens, although the underlying mechanism of action is unknown. Here, we investigated the bactericidal mechanism of PBT2 in the bovine mastitis-causing pathogen, Streptococcus uberis. In this work, we show that PBT2 functions as a Zn^2+^/H^+^ ionophore, exchanging extracellular zinc for intracellular protons in an electroneutral process that leads to cellular zinc accumulation. Zinc accumulation occurs concomitantly with manganese depletion and the production of reactive oxygen species (ROS). PBT2 inhibits the activity of the manganese-dependent superoxide dismutase, SodA, thereby impairing oxidative stress protection. We propose that PBT2-mediated intracellular zinc toxicity in *S. uberis* leads to lethality through multiple bactericidal mechanisms: the production of toxic ROS and the impairment of manganese-dependent antioxidant functions. Collectively, these data show that PBT2 represents a new class of antibacterial ionophores capable of targeting bacterial metal ion homeostasis and cellular redox balance. We propose that this novel and multitarget mechanism of PBT2 makes the development of cross-resistance to medically important antimicrobials unlikely.

**IMPORTANCE** More antimicrobials are used in food-producing animals than in humans, and the extensive use of medically important human antimicrobials poses a significant public health threat in the face of rising antimicrobial resistance. Therefore, the elimination of antimicrobial crossover between human and veterinary medicine is of great interest. Unfortunately, the development of new antimicrobials is an expensive high-risk process fraught with difficulties. The repurposing of chemical agents provides a solution to this problem, and while many have not been originally developed as antimicrobials, they have been proven safe in clinical trials. PBT2, a zinc ionophore, is an experimental therapeutic that met safety criteria but failed efficacy checkpoints against both Alzheimer’s and Huntington’s diseases. It was recently found that PBT2 possessed potent antimicrobial activity, although the mechanism of bacterial cell death is unresolved. In this body of work, we show that PBT2 has multiple mechanisms of antimicrobial action, making the development of PBT2 resistance unlikely.

## Abstract (precis)

More antimicrobials are used in food-producing animals than in humans, and the extensive use of medically important human antimicrobials poses a significant public health threat in the face of rising antimicrobial resistance. Therefore, the elimination of antimicrobial crossover between human and veterinary medicine is of great interest. Unfortunately, the development of new antimicrobials is an expensive high-risk process fraught with difficulties. The repurposing of chemical agents provides a solution to this problem, and while many have not been originally developed as antimicrobials, they have been proven safe in clinical trials. PBT2, a zinc ionophore, is an experimental therapeutic that met safety criteria but failed efficacy checkpoints against both Alzheimer’s and Huntington’s diseases. It was recently found that PBT2 possessed potent antimicrobial activity, although the mechanism of bacterial cell death is unresolved. In this body of work, we show that PBT2 has multiple mechanisms of antimicrobial action, making the development of PBT2 resistance unlikely.

## INTRODUCTION

The widespread emergence of antimicrobial resistance (AMR) threatens the security of modern medicine. Antimicrobial use in food-producing animals is recognized as an important driver of AMR, raising concerns in light of the considerable overlap of antimicrobials used in both humans and animals (1). With food consumption rising to meet the demands of a growing global population, antimicrobial use in food-producing animals is consequently set to increase (2). This intensification of antimicrobial use comes with a greater risk of resistance emerging (3, 4) and warrants the elimination of antimicrobial crossover between human and veterinary medicine.

Bovine mastitis, an inflammatory disease of the bovine mammary gland, is the leading cause for antimicrobial use in the dairy industry worldwide (5, 6). The Gram-positive pathogen Streptococcus uberis is a common environmental cause of bovine mastitis (7). Due to the ubiquity of environmental pathogens such as *S. uberis* in the dairy environment, prevention of environmental mastitis is particularly challenging and relies heavily on antimicrobial sanitizers (8, 9). Chlorhexidine and iodine, which the World Health Organization (WHO) recognizes as essential antiseptics for human medicine (10), are among the most common antiseptics used in teat disinfectants (11). Increased tolerance to chlorhexidine has been reported in both Gram-positive and Gram-negative hospital-associated pathogens (12–14), illustrating the pressing need to discover novel animal-only antimicrobials for mastitis prevention and treatment.

Ionophores represent a class of molecules capable of binding and transporting protons or other cations across biological membranes (15). With the exception of the mycobactericidal drug bedaquiline, with recently demonstrated H^+^/K^+^ ionophoric activity (16), ionophoric drugs (e.g., monensin, lasalocid, salinomycin, and narasin) are exclusively used in agriculture (17). Despite the routine use of ionophores in agriculture for more than 35 years, there is little indication of increasing resistance to these drugs or of cross-resistance to medically important classes of antimicrobials (17). Developing novel antimicrobials with ionophoric mechanisms of action for veterinary-only medicine satisfies the requirement for antimicrobials in food-producing animals without risking crossover with human medicine.

PBT2, a derivative of the 8-hydroxyquinoline (8-HQ) scaffold, has been demonstrated to act as a zinc and copper ionophore in mammalian cells (18). PBT2 facilitates the intracellular accumulation of these metals and showed promise as a therapeutic for targeting the abnormal metallochemistry in Alzheimer’s and Huntington’s diseases (18, 19). In phase II clinical trials, however, efficacy endpoint criteria were not met in PBT2-treated patients with these neurodegenerative diseases (20–22). Despite this, extensive preclinical and clinical data demonstrate PBT2 is safe and well tolerated in humans and in animal (mouse) models (20–22).

In bacterial cells, zinc is an essential micronutrient for normal physiology yet can mediate significant toxicity in excess (23, 24). The essentiality and toxicity of zinc in bacterial pathogens has been exploited by host defense mechanisms that starve cells of zinc, in a concept termed “nutritional immunity” (25, 26), or deliver the metal in excess toxic quantities (27, 28). Given the vulnerability of bacterial cells to zinc stress and the safety profile of PBT2 in mammalian hosts, the antimicrobial potential of this zinc ionophore was recently investigated. Indeed, PBT2 in combination with zinc (PBT2-zinc) has potent antibacterial activity against multiple Gram-positive pathogens (29). Antibacterial activity was observed *in vitro* and *in vivo* in a murine wound infection model, highlighting the potential of PBT2 to be repurposed as a topical antimicrobial in veterinary medicine (29).

While PBT2 was shown to increase cytosolic zinc and disrupt metal ion homeostasis in bacteria (29), a defined molecular mechanism of killing remained unresolved and is key to the development of effective next-generation derivatives. In this study, we show that PBT2 is a Zn^2+^/H^+^ ionophore and exerts bactericidal activity in *S. uberis* through intracellular zinc toxicity, which leads to the accumulation of toxic reactive oxygen species (ROS) and dysregulates manganese homeostasis, causing cells to become hypersensitive to oxidative stress. Our work builds on the current knowledge of the coordinated relationship between zinc and manganese in bacteria and illustrates how it can be exploited for antimicrobial potential.

## RESULTS

### PBT2 and zinc exhibit antibacterial synergy.

**FIG 1:**
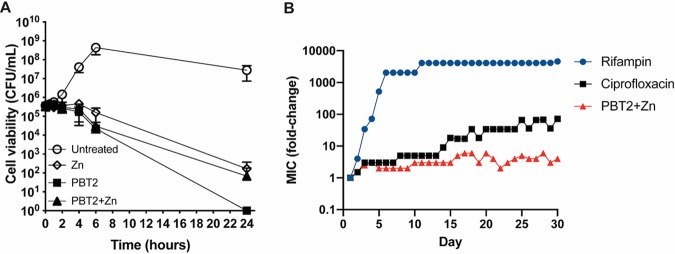
Bactericidal action and resistance development of PBT2 and zinc against *S. uberis*. (A) Time-dependent cell killing of *S. uberis* in response to MICs of PBT2 and zinc, alone (5.0 mg/liter PBT2, 800 μM zinc) and in combination (0.5 mg/liter PBT2 + 10 μM zinc). THB contains a background concentration of 23 μM zinc. Error bars represent the standard deviations of the means from biological triplicates. (B) Development of resistance to PBT2+Zn compared to that for rifampin and ciprofloxacin. *S. uberis* was serially passaged for 30 days in the presence of subinhibitory concentrations of PBT2 (+100 μM Zn), rifampin, or ciprofloxacin in THB. Data represent the means from two biological replicates.

We examined if the reported antibacterial activity of PBT2 extended to the bovine pathogen *S. uberis*. A checkerboard assay was undertaken to determine the MIC and combined inhibitory concentrations (CICs) of PBT2 and zinc. Both PBT2 and zinc individually displayed antibacterial activity against *S. uberis*, with MICs of 5.0 mg/liter (14.5 μM) and 800 μM, respectively (Table S1; see also Fig. S1 in the supplemental material). Combining PBT2 and zinc dramatically increased the antibacterial effectiveness of both compounds, with a CIC of 0.5 mg/liter PBT2 (1.45 μM) in the presence of 10 μM zinc or 0.05 mg/liter PBT2 (0.145 μM) with 100 μM zinc. The fractional inhibitory concentration indexes (FICIs) of 0.113 and 0.135 indicate PBT2 and zinc interact synergistically (FICI ≤ 0.5). A time-dependent cell-killing assay revealed >3-log_10_ reductions in CFU/ml of initial inocula following treatment with the MIC of either PBT2 or zinc, indicating a bactericidal mechanism of action (Fig. 1A). The observed bactericidal activity of PBT2 alone may be attributable to the concentration of 23 μM zinc in the growth medium, Todd-Hewitt broth (THB), which was determined by inductively coupled plasma mass spectrometry (ICP-MS). However, the extent to which PBT2 mobilizes the bioavailable proportion of medium zinc was not determined. Remarkably, treatment with the CIC of PBT2 and zinc (PBT2+Zn) was bactericidal, even at 10-fold and 80-fold lower concentrations of PBT2 and zinc MICs, respectively (Fig. 1A).

10.1128/mSphere.00157-20.1 FIG S1 Checkerboard analysis of PBT2 and Zn synergy. Growth of *S. uberis* NZ01 in response to various concentrations of PBT2 and Zn, alone and in combination, in a checkerboard broth microdilution assay. After 24 h of incubation, growth (OD_600_) was determined by spectrometric measurement on a microplate reader. Data represent the means from biological triplicates. Download FIG S1, TIF file, 0.3 MB. Copyright © 2020 Harbison-Price et al. 2020 Harbison-Price et al. This content is distributed under the terms of the Creative Commons Attribution 4.0 International license.

10.1128/mSphere.00157-20.9 TABLE S1 PBT2 and zinc exhibit antibacterial synergy against *S. uberis.* CIC, combined inhibitory concentration; FIC, fractional inhibitory concentration; FICI, fractional inhibitory concentration index. Download Table S1, DOCX file, 0.01 MB. Copyright © 2020 Harbison-Price et al. 2020 Harbison-Price et al. This content is distributed under the terms of the Creative Commons Attribution 4.0 International license.

We next sought to compare the ability of *S. uberis* to develop resistance to PBT2+Zn and two medically important antibiotics, rifampin and the fluoroquinolone antibiotic ciprofloxacin, by serial passaging through progressively increasing drug concentrations over a course of 30 days. While the ciprofloxacin and rifampin MICs increased by 72-fold and 4,608-fold, respectively, the PBT2+Zn MIC increased by only 4-fold (Fig. 1B).

Because antimicrobial activity can be negatively affected by proteins and lipids present in milk (30), assessing the antimicrobial efficacy in bovine milk is important for the development of potential new therapeutics for mastitis prevention or treatment. We tested the bactericidal efficacy of PBT2+Zn in whole cow’s milk and observed that PBT2 remained bactericidal against *S. uberis* (MBCs of 32 mg/liter for PBT2 + 0 μM Zn, 16 mg/liter for PBT2 + 200 μM Zn, and 8 mg/liter for PBT2 + 400 μM Zn).

### PBT2-zinc disrupts metal ion homeostasis.

**FIG 2:**
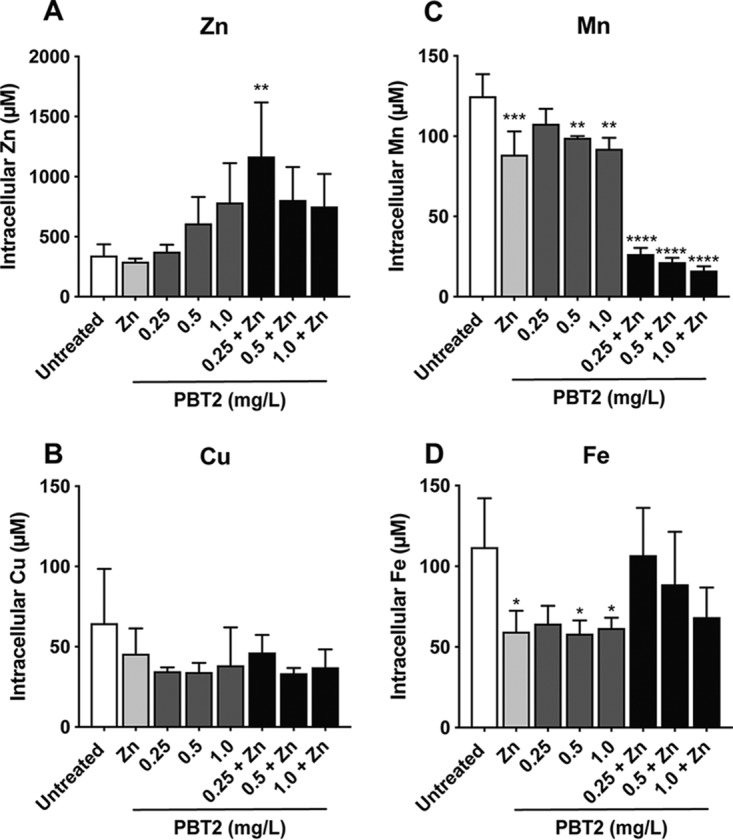
PBT2-zinc alters intracellular metal ion homeostasis. Intracellular zinc (A), copper (B), manganese (C), and iron (D) concentrations as determined by ICP-MS of mid-log-phase *S. uberis* NZ01 cells (OD_600_ of 0.3) treated with various concentrations of PBT2 with or without additional zinc (100 μM) or untreated. Error bars represent the standard deviations of the means from biological triplicates. *, *P* < 0.05; **, *P* < 0.005; ***, *P* < 0.001; ****, *P* < 0.0001 by one-way analysis of variance (ANOVA).

ICP-MS analysis was used to determine the metal ion content of *S. uberis* cells in response to PBT2 treatment. Consistent with the ionophore activity of PBT2, a concentration-dependent increase in whole-cell zinc accumulation was observed in response to increasing concentrations of PBT2 (Fig. 2A). While 0.25 mg/liter PBT2 alone had no effect on cellular zinc abundance, in the presence of zinc (100 μM), cells treated with 0.25 mg/liter PBT2 accumulated >3-fold more zinc than untreated cells (*P* = 0.006) (Fig. 2A). Additional PBT2 did not increase cellular zinc further, with zinc plateauing at a similar level to that in cells treated with the highest tested concentration of PBT2 alone (1.0 mg/liter) (Fig. 2A). This suggests zinc accumulated by higher concentrations of PBT2 may exceed tolerated cellular levels and either induces mechanisms to maintain zinc homeostasis or causes cell death. Previous evidence in mammalian cell models indicates that PBT2 acts as both a zinc and copper ionophore (18). However, there were no observable changes in *S. uberis* copper levels in response to PBT2 treatment (Fig. 2B). This is likely attributable to the low concentration of copper (1 μM) in the growth medium relative to that of zinc (23 μM). We speculate that under our experimental conditions, zinc outcompetes copper for chelation to PBT2.

Treatment with PBT2 and zinc altered the cellular abundances of manganese and iron (Fig. 2C and D). Manganese levels decreased by up to 7.5-fold in response to PBT2-zinc challenge (*P* = 0.0001) compared to those in untreated cells (Fig. 2C). Decreased manganese levels were also observed in cells treated with either PBT2 or zinc alone (Fig. 2C). Exogenous zinc was previously reported to deplete cellular manganese in Streptococcus pneumoniae, causing cells to become hypersensitive to oxidative stress (27, 31). In response to oxidative stress, streptococci restrict cellular iron levels as a strategy to limit the generation of ROS through iron-mediated Fenton chemistry (32–34). Similarly, *S. uberis* treated with either PBT2 (0.5 and 1.0 mg/liter) or zinc had significantly lower cellular iron than untreated cells (*P* = 0.0387, 0.0335, and 0.0498, respectively) (Fig. 2D). Collectively, these data suggest that PBT2-mediated zinc accumulation perturbs normal metal ion homeostasis and may sensitize cells to oxidative stress.

### PBT2 exchanges zinc ions for protons.

Ionophores are compounds with the ability to translocate cations, protons, or both and are therefore capable of dissipating either the membrane potential (Δψ) (e.g., valinomycin) or transmembrane pH gradient (ΔpH) (e.g., nigericin) components of the proton motive force (PMF) (15). To examine if the bactericidal mechanism of PBT2 is explained by dissipation of the PMF, we measured the Δψ and ΔpH of *S. uberis* cells in response to PBT2-zinc by using the radioisotopes [^14^C]methyltriphenyl phosphonium iodide ([^14^C]TPP^+^) and [7-^14^C]benzoate, respectively. No observable differences in either component of the PMF occurred in response to treatment with PBT2-zinc at either 1× or 10× the CIC (0.5 mg/liter PBT2 + 10 μM zinc) (see Fig. S2).

10.1128/mSphere.00157-20.2 FIG S2 Effect of PBT2-zinc on components of the protonmotive force (PMF). Internal pH (A), transmembrane pH gradient (ΔpH) (B), and membrane potential (ΔΨ) (mV) (C) of *S. uberis* cells following treatment with PBT2-Zn at 1× CIC (0.5 mg/liter PBT2 + 10 μM zinc) or 10× CIC (5.0 mg/liter PBT2 + 100 μM zinc) for 20 min. As a control, cells were treated with toluene (0.4% [vol/vol]) to permeabilize cell membranes and dissipate the ΔpH and ΔΨ. In panels A and B, cells were suspended in THB at pH 5.2 to establish a large ΔpH, while cells in panel C were suspended in THB at pH 7.5. The ΔpH was calculated from the distribution of [^14^C]benzoate using the Henderson-Hasselbalch equation, and the ΔΨ was determined from the uptake of [^14^C]TPP^+^ according to the Nernst relationship. Internal pH was determined from the ΔpH. Error bars represent the standard deviations of the mean from a biological triplicate (ns, *P* > 0.05; ****, *P* < 0.0001, one-way ANOVA). Download FIG S2, TIF file, 0.3 MB. Copyright © 2020 Harbison-Price et al. 2020 Harbison-Price et al. This content is distributed under the terms of the Creative Commons Attribution 4.0 International license.

**FIG 3:**
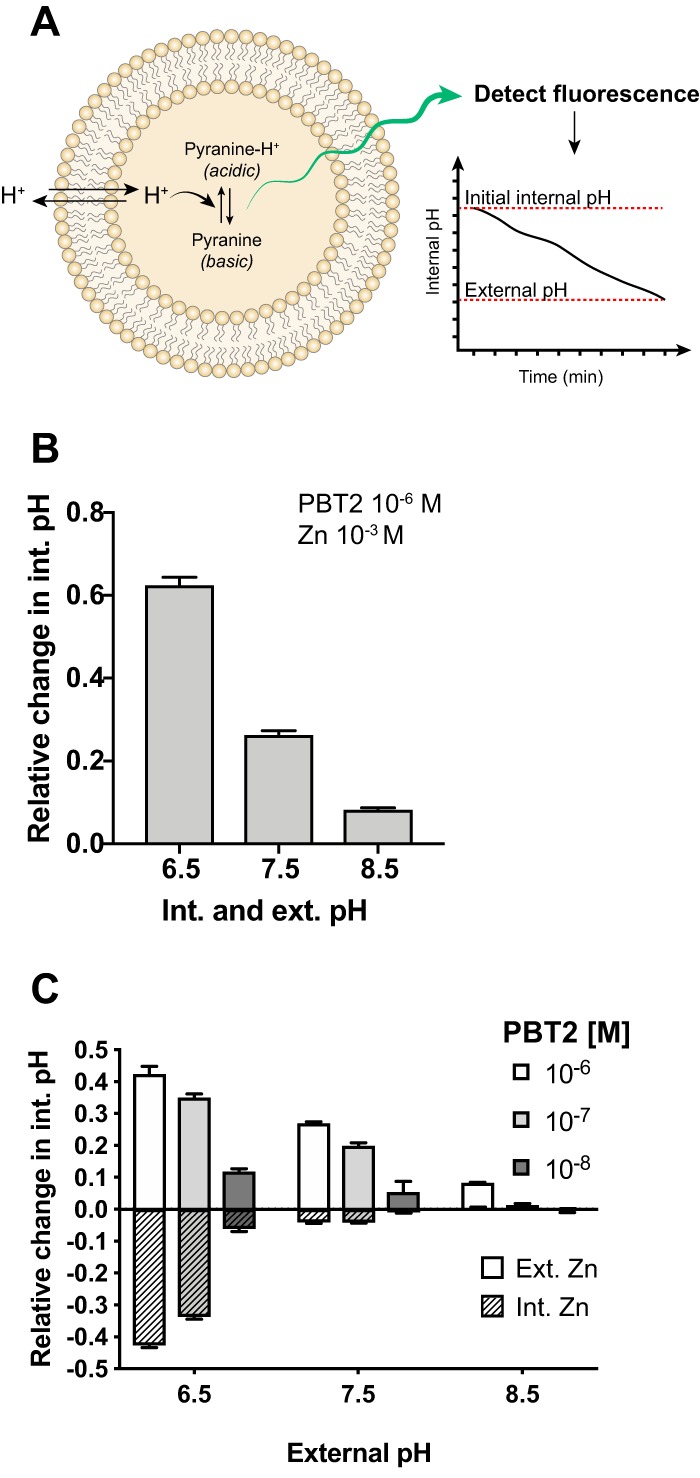
PBT2 is a Zn^2+^/H^+^ ionophore. (A) Schematic for measuring internal pH using pyranine-containing liposomes: pyranine is a non-membrane-permeable pH indicator where the ratio of the acidic to basic forms of pyranine is pH dependent. Excitation of acidic (λ_ex_ 405 nm) and basic (λ_ex_ 455 nm) pyranine generates detectable fluorescence (λ_em_ 510 nm). The internal pH of liposomes equilibrates over time with the external pH, and internal pH can be determined using a standard curve of pH to pyranine fluorescence ratios. In these experiments, pyranine fluorescence was measured and internal pH was determined after addition of PBT2 and/or zinc to liposomes from a 15-min endpoint. Changes to internal pH by PBT2-zinc were normalized for the effect of external zinc (10^−3^ M) alone. (B) Changes in internal pH of pyranine-containing liposomes with matching internal and external pH values following addition of PBT2 (10^−6^ M) in combination with zinc (10^−3^ M). (C) Relative changes in internal pHs of liposomes suspended in Mes-Mops-Tris buffer (pH indicated) containing PBT2 (10^−6^, 10^−7^, or 10^−8^ M), where zinc (10^−3^ M) is either external to liposomes (plain bars) or internal (striped bars). Error bars represent the standard deviations from triplicate measurements.

As we observed an increase in cellular zinc, but no effect on the membrane potential of whole cells (Fig. S2C), we hypothesized that a counterion (either protons or other cations) might be moved to make the process electroneutral. To test this, we assessed the ability of PBT2 to move protons in phosphatidylcholine liposomes loaded with the non-membrane-permeable and pH-sensitive probe, pyranine (Fig. 3). Pyranine-containing liposomes have previously been used as a controlled system for measuring internal pH changes in response to protonophoric or ionophoric drugs (16, 35) where finite, (electro)chemical gradients are artificially established and used to assess proton movement across artificial lipid bilayers. Changes in the liposome luminal pH (internal pH) are detected by measuring the fluorescence of the acidic and basic forms of pyranine, the ratio of which is dependent on pH (Fig. 3A).

In response to external zinc addition, PBT2 was able to cause a concomitant increase (alkalization) of the liposome internal pH (Fig. 3B and C and Fig. S3). As PBT2-zinc does not alter the cell membrane permeability of group A *Streptococcus* (GAS), vancomycin-resistant *Enterococcus* (VRE), or methicillin-resistant Staphylococcus aureus (MRSA) (29), we attribute this effect to a direct interaction of PBT2 with protons rather than proton leakage through damaged liposomal membranes. This effect was not observed in the absence of zinc (see Fig. S4), suggesting it is dependent on the formation of a PBT2-zinc complex. Using isothermal titration calorimetry (ITC), we confirmed the formation of 2:1 PBT2-Zn complexes, as previously observed (see Fig. S5) (36). Collectively this suggests PBT2 exchanges zinc for protons in an antiport-like process. Inverting the gradient, by incorporating zinc inside the liposome during preparation, resulted in a concomitant internal acidification upon PBT2 addition (Fig. 3C and Fig. S6). This suggests the process is not direction specific and is driven purely by the Zn^2+^ concentration gradient. Notably, acidification of the assay buffer to pH 6.5 enhanced this antiport activity up to 7.5-fold, compared to that at pH 8.5 (Fig. 3B and C and Fig. S3). Given the apparent absence of a biological effect from PBT2-mediated proton translocation, we continued this work with a focus on the consequences of zinc delivery into cells by PBT2.

10.1128/mSphere.00157-20.3 FIG S3 Effects of PBT2-zinc on internal pH of empty liposomes. Pyranine-containing liposomes with an internal pH of 6.5 (A), 7.7 (B), or 8.5 (C) were suspended in MES-MOPs-Tris buffer of matching pH and treated with PBT2 and zinc. Actual measured internal pH values of liposomes, including untreated and vehicle (DMSO)-treated controls, are shown on the left. The relative change in internal pH of liposomes treated with PBT2 is shown on the right and is normalized for the effect of zinc alone on internal pH. Error bars represent the standard deviations from triplicate measurements. Download FIG S3, TIF file, 0.4 MB. Copyright © 2020 Harbison-Price et al. 2020 Harbison-Price et al. This content is distributed under the terms of the Creative Commons Attribution 4.0 International license.

10.1128/mSphere.00157-20.4 FIG S4 Effect of PBT2 on internal pH of liposomes in the absence of zinc. Pyranine-containing liposomes with an initial internal pH of 6.5, 7.7, or 8.5 were suspended in buffer of matching pH and treated with various concentrations of PBT2. Untreated and vehicle (DMSO) controls are indicated. Error bars represent the standard deviations from triplicate measurements. Download FIG S4, TIF file, 0.2 MB. Copyright © 2020 Harbison-Price et al. 2020 Harbison-Price et al. This content is distributed under the terms of the Creative Commons Attribution 4.0 International license.

10.1128/mSphere.00157-20.5 FIG S5 PBT2 binds zinc in a 2:1 stoichiometry. Titration curve and binding isotherm of 0.3 mM zinc injected into 0.035 mM PBT2 at pH 7.7 and 37°C, with best-fit thermodynamic parameter estimates of K = 1.97 × 10^−6^ ± 3.12 × 10^−5^ M and *ΔH* = −7.48 ± 0.14 kcal/mol. Download FIG S5, TIF file, 0.1 MB. Copyright © 2020 Harbison-Price et al. 2020 Harbison-Price et al. This content is distributed under the terms of the Creative Commons Attribution 4.0 International license.

10.1128/mSphere.00157-20.6 FIG S6 Effects of PBT2 on internal pH of zinc-containing liposomes. (A) Internal pH of untreated zinc-containing liposomes following suspension in buffer at pH 6.5, 7.5, or 8.5. Initial internal pH of liposome preparations was pH 7.5. (B) Internal pH of pyranine-containing liposomes loaded with zinc (10^−3^ M) following suspension in buffer at pH 6.5, 7.7, or 8.5 with various concentrations of PBT2. (C) Change in internal pH of zinc-containing liposomes suspended in buffer of pH 6.5, 7.7, or 8.5 with various concentrations of PBT2, relative to internal pH of untreated controls. Error bars represent the standard deviations from triplicate measurements. Download FIG S6, TIF file, 0.2 MB. Copyright © 2020 Harbison-Price et al. 2020 Harbison-Price et al. This content is distributed under the terms of the Creative Commons Attribution 4.0 International license.

### PBT2-zinc induces transcriptional changes to metal ion transporter genes.

**FIG 4:**
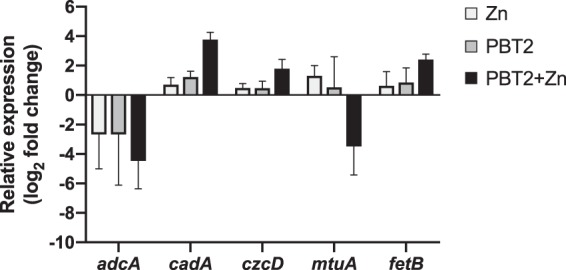
PBT2-zinc alters the expression of metal ion transport genes. Relative expression of selected genes in mid-log-phase *S. uberis* cells (OD_600_ of 0.3) after 1 h of treatment with or without PBT2 (1.0 mg/liter) and zinc (100 μM), individually or in combination. Relative expression (expressed as log_2_-fold change) was calculated relative to the untreated control and normalized to the reference gene (*pflC*) using the *ΔΔC_T_* method. Error bars represent the standard deviations of the means from biological triplicates.

To further understand the effect of PBT2 and zinc on metal ion homeostasis, changes in the expression of genes involved in metal ion transport were investigated. Consistent with the observed changes in metal ion levels, several metal ion transporters were differentially expressed in response to PBT2-zinc challenge (Fig. 4). *S. uberis* contains the ATP-binding cassette (ABC) permease AdcABC, a conserved zinc acquisition system among streptococci (37). We observed that the zinc-specific solute binding protein (SBP), *adcA* (CGZ53_03345), was downregulated 4.5 log_2_-fold in response to PBT2-zinc (Fig. 4). Consistent with previous work on *cadA* homologs demonstrating function in zinc export, *S. uberis cadA* (CGZ53_06375) was upregulated 3.7 log_2_-fold in response to PBT2-zinc (Fig. 4). In another demonstration of increased zinc export in response to PBT2-zinc, expression of a putative homolog of the *czcD* zinc efflux pump (CGZ53_05790) increased 1.8 log_2_-fold (Fig. 4).

*S. uberis* contains a homolog of the S. pneumoniae PsaABC manganese importer, termed MtuABC, with an established role in manganese uptake (38). Consistent with the observed depletion in manganese upon PBT2-zinc treatment (Fig. 2C), transcriptional profiling revealed that *mtuA* (CGZ53_02610) was strongly downregulated (3.5 log_2_-fold) in response to PBT2-zinc treatment (Fig. 4). The upregulation of *fetB*, a putative iron export ABC transporter permease, following PBT2-zinc treatment, together with our previous finding of decreased cellular iron in PBT2-zinc treated wild-type cells (Fig. 4 and 2D), supports the inference that cells must tightly regulate iron to avoid iron toxicity under conditions where antioxidative mechanisms are compromised.

### PBT2-mediated zinc toxicity sensitizes cells to killing by ROS.

**FIG 5:**
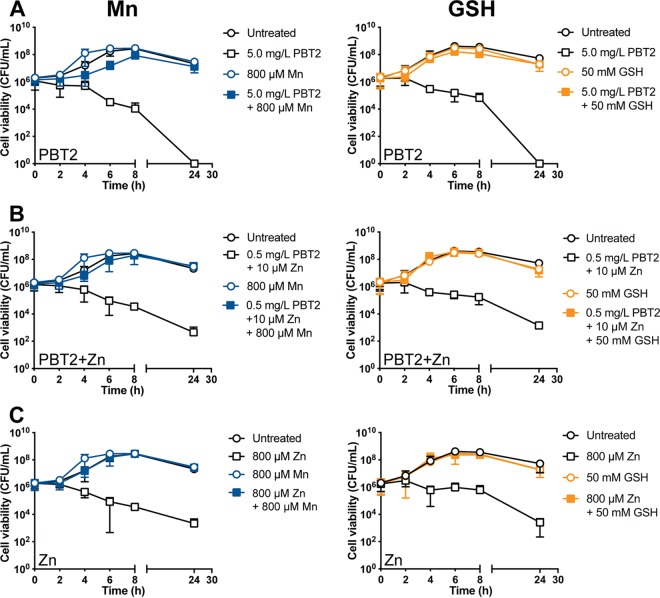
Manganese and reduced glutathione (GSH) protect against PBT2-zinc killing. Pretreatment of *S. uberis* cells (OD_600_ of 0.05) with manganese or GSH before the addition of PBT2 (A), zinc (B), or PBT2+Zn (C) at respective MICs and CICs prevents cell killing. Error bars represent the standard deviations of the mean from biological triplicates.

If excess zinc in *S. uberis* exerts toxicity through manganese starvation, as previously observed in S. pneumoniae (27), then supplementing cells with manganese should rescue the observed lethality associated with PBT2-zinc treatment. We found cultures pretreated with manganese (800 μM) were completely protected against cell killing by PBT2, zinc, and PBT2-zinc (Fig. 5A to C).

Manganese has prominent roles in oxidative stress protection in biological systems, from serving as a cofactor for manganese-dependent superoxide dismutase to forming ROS-scavenging Mn-organic acid complexes (39–41). As in related streptococcal species (31, 33), *S. uberis* relies solely on a manganese-dependent superoxide dismutase (SodA) for the conversion of accumulated O_2_·^−^ to H_2_O_2_ and O_2_. To determine if oxidative stress contributes to the lethality of PBT2 and zinc, we tested whether an antioxidant could prevent cell killing. Pretreatment with reduced glutathione (GSH) (50 mM) added extracellularly, an antioxidant capable of neutralizing ROS in bacteria (42), provided protection against the bactericidal action of PBT2 and zinc (Fig. 5A to C). Because zinc is able to bind to the sulfhydryl group of the GSH cysteine moiety and form GSH-Zn complexes (43), which may explain the observed protective effect of GSH, we undertook further experimentation to clarify the role of oxidative stress in the PBT2-zinc bactericidal mechanism.

**FIG 6:**
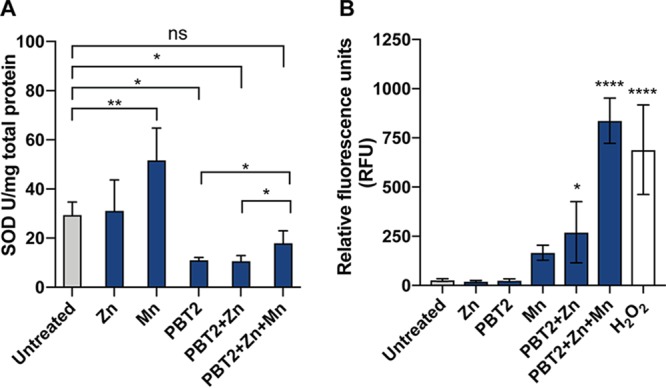
PBT2-zinc impairs SodA activity and generates ROS. (A) Superoxide dismutase (SOD) activity of mid-log-phase *S. uberis* cells (OD_600_ of 0.3) treated with PBT2 (1.0 mg/liter), zinc (100 μM), or manganese (800 μM), individually or in combination. Cell lysates were analyzed for SOD activity and normalized for total protein. (B) H_2_O_2_ generation of mid-log-phase *S. uberis* cells (OD_600_ of 0.3) treated with PBT2 (1.0 mg/liter), zinc (100 μM), or manganese (800 μM), individually or in combination, as detected by Amplex Red fluorescence (560 λ_ex_, 590 λ_em_). Untreated and H_2_O_2_-treated controls are included for comparison. Error bars represent the standard deviations of the means from biological triplicates. ns, *P* > 0.05; *, *P* < 0.05; **, *P* < 0.005; ***, *P* < 0.001; ****, *P* < 0.0001 by one-way ANOVA.

We hypothesized that by depleting intracellular manganese, PBT2-zinc would inhibit SodA enzyme activity. As anticipated, cultures treated with PBT2-zinc showed a 2.7-fold reduction in SodA activity compared to that in untreated cells (*P* = 0.0156) (Fig. 6A). Treatment with PBT2 alone reduced SodA activity by 2.6-fold (*P* = 0.0179), but zinc alone had no effect (Fig. 6A). Transcriptional profiling of *sodA* revealed no changes in expression following PBT2, zinc, or PBT2-zinc treatment, suggesting PBT2 does not interfere with normal *sodA* regulation (see Fig. S7). Provision of excess exogenous manganese significantly increased the SodA activity (*P* = 0.0041), and when in combination with PBT2-zinc, manganese partially restored SodA function (*P* = 0.0345) (Fig. 6A).

10.1128/mSphere.00157-20.7 FIG S7 Expression of oxidative-stress response genes following PBT2 and zinc treatment. Relative expression of *ahpF*, *ahpC*, and *sodA* genes in mid-log-phase *S. uberis* cells (OD_600_ of 0.3) after 1 h treatment with or without PBT2 (1.0 mg/liter) and zinc (100 μM), individually or in combination. Relative expression (expressed as log_2_-fold change) was calculated relative to the untreated control and normalized to the reference gene (*pflC*) using the *ΔΔC_T_* method. Error bars represent the standard deviations of the means from biological triplicates. Download FIG S7, TIF file, 0.1 MB. Copyright © 2020 Harbison-Price et al. 2020 Harbison-Price et al. This content is distributed under the terms of the Creative Commons Attribution 4.0 International license.

To examine if ROS accumulate as a consequence of PBT2-mediated zinc toxicity and manganese starvation, we specifically measured the production of H_2_O_2_. We found that PBT2 or zinc alone had no effect on H_2_O_2_ levels compared to that in untreated cells (Fig. 6B). However, cells treated with PBT2-zinc showed increased H_2_O_2_ (*P* = 0.0380) (Fig. 6B). *S. uberis* harbors an alkyl hydroperoxidase (*ahpC* and *ahpF*) for the detoxification of H_2_O_2_, yet no changes in expression of either *ahpC* or *ahpF* were observed in response to PBT2-zinc (Fig. S7). As a critical test of the hypothesis that manganese abolishes PBT2-zinc lethality by restoring antioxidant function, we examined the effect of manganese on H_2_O_2_ production. The addition of exogenous manganese to PBT2-zinc-treated cells dramatically increased detectable H_2_O_2_ (*P* = 0.0001) (Fig. 6B). This finding supports the notion that providing excess manganese enables the sole superoxide dismutase, the manganese-dependent SodA, to convert accumulated O_2_·^−^ to H_2_O_2_ and O_2_, ultimately increasing detectable H_2_O_2_. Treatment with manganese alone also demonstrated an increase in H_2_O_2_, although this was not statistically significant (*P* = 0.4742) (Fig. 6B). This supports an increased rate of O_2_·^−^ turnover through SodA by provision of excess amounts of the manganese cofactor.

## DISCUSSION

In this work, we show that the bactericidal mechanism of PBT2 is mediated by intracellular zinc toxicity, which disrupts manganese homeostasis and leads to the accumulation of toxic ROS. In this global bactericidal mechanism of action, *S. uberis* was unable to generate significant resistance to PBT2+Zn in comparison to the single-target antibiotics ciprofloxacin (44) and rifampin (45). In addition, this work provides a molecular mechanism for PBT2 ionophoric activity in bacterial cells, whereby PBT2 translocates across the bacterial cell membrane acting as a Zn^2+^/H^+^ ionophore. The absence of an effect on the membrane potential indicates PBT2 functions as an electroneutral ionophore, in support of an exchange of one Zn^2+^ ion for two H^+^ ions. While there was a pronounced alkalization of pyranine-containing liposomes from PBT2-mediated proton translocation, no changes in internal pH were found in whole cells. Bacterial cells employ multiple homeostatic mechanisms to maintain intracellular pH in a narrow tolerable range (46), and such regulatory mechanisms likely protect cells from intracellular pH alterations by PBT2.

**FIG 7:**
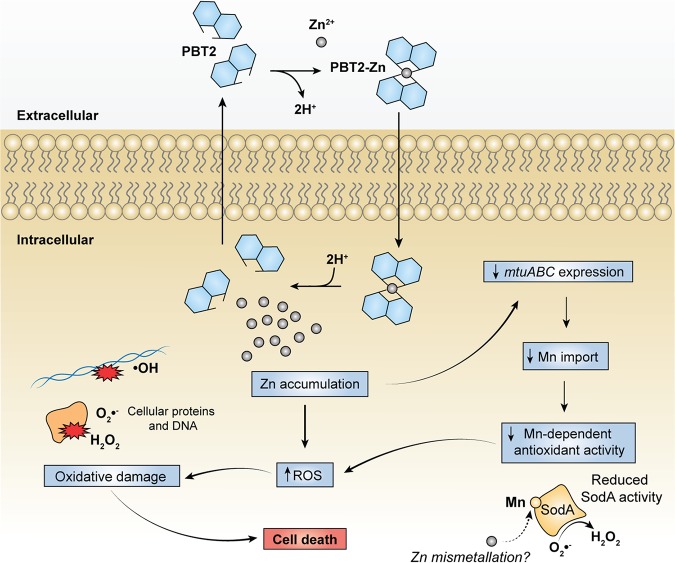
Proposed bactericidal model of action of PBT2. PBT2 has Zn^2+^/H^+^ ionophore activity and mediates intracellular zinc accumulation by exchanging extracellular Zn^2+^ for intracellular 2H^+^ in an electroneutral process. Excess zinc leads to ROS accumulation and additionally dysregulates manganese homeostasis by inappropriately downregulating expression of the MtuABC manganese import system. The resulting cellular manganese depletion predisposes cells to oxidative stress by limiting manganese-dependent antioxidant activity. The manganese-dependent SodA may be inhibited by two mechanisms: depletion of the essential manganese cofactor and zinc mismetallation. Cell death results when ROS accumulate beyond a tolerable threshold and induce excessive oxidative damage to essential cellular proteins and genomic DNA.

We observed an enhancement of PBT2 ionophoric activity at lower pH, suggesting that protonation of PBT2 is required for efficient zinc transport. A detailed structural analysis by Nguyen et al. (36) has revealed zinc chelation by PBT2 occurs through the pyridine nitrogen and deprotonated phenoxide. Protonation of the PBT2-zinc complex at low pH may therefore promote the release of zinc according to the membrane potential and zinc concentration gradient. Given that cytosolic “free” zinc in bacterial cells is in the picomolar to nanomolar range (47) and PBT2 binds zinc with an affinity of ∼2 μM, it is unlikely PBT2 is capable of moving zinc ions out of bacterial cells. Collectively, these data, along with the finding of PBT2-mediated zinc accumulation, provide a novel molecular model for PBT2 ionophoric activity in which PBT2 exchanges extracellular zinc for intracellular protons in an electroneutral process (Fig. 7).

Similar to previous work investigating zinc toxicity in S. pneumoniae (27, 31, 48), we show exogenous zinc significantly depletes cellular manganese in *S. uberis*. Consistently, addition of manganese (800 μM) protects against zinc toxicity. In these previous studies, the proposed mechanism of zinc-mediated manganese starvation is through zinc competitively binding to the extracellular PsaA, the manganese SBP of the PsaABC manganese importer, thereby limiting manganese uptake. Given the identical compositions of the metal-binding sites in the *S. uberis* PsaA homolog (MtuA) and S. pneumoniae PsaA (38), zinc may similarly impede manganese import in *S. uberis* by mismetallation of MtuA. While this may be the case under conditions of excess exogenous zinc, manganese depletion occurred concomitantly with PBT2-mediated intracellular zinc accumulation. This indicates the existence of an alternative mechanism whereby intracellular zinc intoxication interferes with normal manganese homeostasis. The depletion of *mtuA* transcripts following PBT2-zinc treatment indicates excess intracellular zinc dysregulates *mtuA* expression, thereby preventing manganese import. Consistently, Bohlmann et al. (29) found that expression of the GAS *mtuA* homolog (*mtsA*) is reduced 2.3 log_2_-fold in response to PBT2-zinc. The molecular target of this zinc mismetallation was not identified in our study; however, mismetallation of metalloregulators, which sense specific metal ions and modulate transcription of regulated genes in accordance with physiological needs, was previously observed under conditions of cytosolic metal intoxication (49). In Bacillus subtilis, intracellular zinc intoxication leads to mismetallation of the peroxide response regulator PerR, which, when bound to zinc, cannot repress heme biosynthesis and consequently leads to toxic heme accumulation (50). Similarly, under conditions of manganese intoxication, the ferric uptake regulator Fur is mismetallated with manganese and inappropriately represses iron uptake (51).

Given that exogenous zinc (100 μM) does not affect intracellular zinc content nor SodA activity yet depletes manganese to similar levels as PBT2 alone, PBT2-mediated zinc accumulation may inhibit SodA function through zinc mismetallation. Reduced SodA activity as a result of the zinc ionophoric activity of PBT2 may therefore result from a combination of depletion of the essential manganese cofactor and mismetallation of SodA with zinc. Mismetallation of the GAS SodA with iron reduces the enzyme activity (33); however, investigations into the functionality of the Zn-SodA enzyme are lacking.

The accumulation of H_2_O_2_ in PBT2-zinc-treated cells indicates a bactericidal mechanism whereby intracellular zinc intoxication leads to the generation of ROS, in addition to impairing the ability to manage oxidative stress. Zinc lacks biological redox activity but may perturb the intracellular redox status indirectly through protein mismetallation and inactivation. Indeed, heme accumulation following zinc mismetallation of PerR causes toxic superoxide generation as a result of redox cycling between heme and menaquinone (50). Because the provision of manganese to PBT2-zinc-treated cells increased cellular H_2_O_2_, we infer that intracellular zinc intoxication accumulates O_2_·^−^ in addition to H_2_O_2_. In addition to providing protection against O_2_·^−^, manganese can catalytically scavenge H_2_O_2_ and replace iron in the active sites of iron-containing enzymes, thereby preventing iron oxidation by H_2_O_2_ and subsequent protein inactivation (52, 53). Our data suggest that providing sufficient manganese restores the activity of Mn-SodA and nonenzymatic manganese-based antioxidants that scavenge ROS, thereby reestablishing redox balance under conditions of zinc toxicity.

We propose a model for the bactericidal mechanism of action of the zinc ionophore PBT2 (Fig. 7): PBT2 binds extracellular zinc as a complex and dissociates in the cytoplasm, releasing zinc in exchange for intracellular protons in an electroneutral process that leads to cellular zinc accumulation. Excess intracellular zinc perturbs normal metal ion homeostasis and dysregulates the expression of *mtuABC*, causing manganese depletion. Zinc intoxication additionally disrupts the intracellular redox balance and ROS accumulate. Antioxidant activity is compromised under these manganese-starved conditions, leaving the cell vulnerable to toxic O_2_·^−^ and H_2_O_2_. Ferrous iron (Fe^2+^) may potentiate this oxidative stress by reacting with H_2_O_2_ in the Fenton reaction, generating damaging hydroxyl radicals (·OH). Extensive oxidative damage to DNA by ·OH and to cellular proteins by O_2_·^−^ and H_2_O_2_ (54) ultimately proves lethal to the bacterial cell.

Here, we have demonstrated the original intended ability of PBT2 in altering the metallochemistry in Alzheimer’s and Huntington’s diseases can be used to destabilize metal ion and redox homeostasis in *S. uberis*, sensitizing cells to oxidative stress in a global bactericidal mechanism. Given the essentiality of manganese acquisition for *S. uberis* to infect the bovine mammary gland (38) and the observed bactericidal activity of PBT2 in bovine milk, the therapeutic potential of PBT2 as an antimicrobial for mastitis prevention or treatment is promising. Indeed, the importance of manganese for virulence extends to other pathogens, including S. pneumoniae, GAS, Staphylococcus aureus, and Bacillus anthracis (27, 55–57). PBT2 represents a new class of antibacterial capable of targeting bacterial metal ion homeostasis, and through understanding the molecular basis of zinc ionophoric toxicity, this work serves as a platform for designing future lead compounds with potential for veterinary-only medicine.

## MATERIALS AND METHODS

### Bacterial strain and growth conditions.

Streptococcus uberis strain NZ01 (58) was isolated from a clinical bovine mastitis case in the Manawatu-Wanganui region of New Zealand. *S. uberis* NZ01 was grown in Todd-Hewitt broth (THB) (Sigma-Aldrich) or on THB agar (THA) containing 1.5% (wt/vol) agar for all experiments. Bacteria were routinely grown at 37°C with agitation (200 rpm). All inoculations were performed from overnight cultures (16 h) to a final optical density at 600 nm (OD_600_) of 0.05.

### Chemical and radiochemical reagents.

PBT2 was synthesized as described previously (29). All stocks of PBT2 (2 mg/ml) were prepared in 100% dimethyl sulfoxide (DMSO) unless otherwise stated. ZnSO_4_·7H_2_O (zinc) and MnSO_4_·H_2_O (manganese) stocks were prepared in filter-sterilized distilled water (dH_2_O). The following radiochemicals were obtained from Moravek Inc.: [7-^14^C]benzoate (54.1 mCi/mmol), [^14^C]methyltriphenyl phosphonium iodide (TPP^+^) (57 mCi/mmol), [^14^C]polyethylene glycol 4000 (PEG), and [^3^H]water (1 mCi/g).

### Determination of cell growth inhibition.

The antibacterial activities of PBT2 and zinc, both alone and in combination, were assessed with a checkerboard broth microdilution assay. Various concentration combinations of PBT2 (0 to 10 mg/liter [0 to 29 μM]) and zinc (0 to 800 μM) were arrayed in a 96-well flat-bottom microtiter plate and resuspended in THB inoculated with *S. uberis* NZ01 (OD_600_ of 0.05) at a final volume of 200 μl. After 24 h of incubation, cell growth (OD_600_) was determined by a Varioskan Flash microplate reader (Thermo Scientific). The PBT2 and zinc MIC and CIC were determined as the lowest concentrations that showed no visible growth. The interaction effects were derived from calculation of the FICI, the sum of the FIC for each compound, which is in turn defined as the quotient of the MIC of the inhibitor in combination (CIC) and the MIC of the inhibitor alone, as follows (59):

$$\text{FICI}={\text{FIC}}_{\text{PBT}2}+{\text{FIC}}_{\text{Zn}}=\left(\frac{{\text{CIC}}_{\text{PBT}2}}{{\text{MIC}}_{\text{PBT}2}}\right)+\left(\frac{{\text{CIC}}_{\text{Zn}}}{{\text{MIC}}_{\text{Zn}}}\right).$$

FICI analysis was interpreted according to threshold values described previously (60), whereby synergism between two inhibitors is regarded at an FICI of ≤0.5, antagonism at an FICI of ≥4.0, and no interaction at an FICI between 0.5 and 4.0. Checkerboard assays were undertaken in biological triplicates.

For susceptibility testing of PBT2 in bovine milk, overnight cultures of *S. uberis* NZ01 were harvested by centrifugation and washed twice in phosphate-buffered saline (PBS) (3,220 × *g* for 10 min at 4°C) before inoculation into pasteurized whole-fat (3.8%) cow milk at an OD_600_ of 0.1. *S. uberis* cells were then inoculated 1:2 into milk containing 2-fold serial dilutions of PBT2 with various concentrations of Zn in a 96-well flat-bottom microtiter plate. After a 24-h incubation at 37°C, 10-μl aliquots were spot plated on THA plates according to the Miles-Misra drop plate method (61). Bactericidal activity was examined after 24 h of incubation at 37°C, in which a >3-log_10_ reduction in CFU/ml was determined as bactericidal. As a measure of sterility, the absence of CFU in uninoculated milk was verified by plating on THA.

### Bacterial time-dependent cell killing assays.

*S. uberis* NZ01 was inoculated at an OD_600_ of 0.05 in THB only, THB containing the MIC of PBT2 (5.0 mg/liter) or zinc (800 μM), or THB containing a CIC of PBT2 and zinc (0.5 mg/liter PBT2 and 10 μM zinc) in biological triplicates. Aliquots of cultures were taken at 0, 2, 4, 6, and 24 h postchallenge, serially diluted in 1× phosphate-buffered saline (PBS), and spot plated on THA plates. The surviving CFU/ml of cultures was determined after a 24-h incubation at 37°C.

### Examination of resistance development.

Serial passaging experiments to generate resistance to PBT2+Zn/antibiotics were undertaken as described in Bohlmann et al. (29). Broth (THB) 2-fold microdilution assays of PBT2 in the presence of 100 μM Zn, ciprofloxacin, and rifampin were first set up in a 96-well flat-bottom microtiter plate with an initial inoculum of *S. uberis* NZ01 at an OD_600_ of 0.05. Cells from the highest PBT2+Zn or antibiotic concentrations that showed growth after 24 h of incubation were subcultured in a new microtiter plate with 2-fold dilutions of PBT2+Zn or antibiotics. Serial passaging was repeated for 30 days in biological duplicates.

### Inductively coupled plasma mass spectrometry.

Overnight cultures of *S. uberis* were diluted to an OD_600_ of 0.05 in THB and grown to mid-log phase (OD_600_ of 0.3). Aliquots (45 ml) of mid-log-phase cells were separated into individual flasks and challenged for 1 h with PBT2 (0.25, 0.5, or 1.0 mg/liter) with or without zinc (100 μM) or untreated. Samples were prepared for ICP-MS analysis according to the protocol used by Bohlmann et al. (29). The metal contents of samples were determined using an Agilent 7500ce ICP-MS (Centre for Trace Element Analysis, Department of Chemistry, University of Otago). Three biological replicates were analyzed. For analysis of THB, a 2-ml aliquot of the medium was sent for analysis.

Metal ion concentrations (milligrams/liter) of samples were converted to molar units, whereby the intracellular metal ion concentrations of bacterial samples were calculated from the *S. uberis* NZ01 protein content (111 mg protein/OD_600_/liter) and internal cell volume (3.76 ± 0.78 μl/mg protein). Protein content was determined by a bicinchoninic acid (BCA) assay, and the internal cell volume was estimated from the difference between the intracellular and extracellular partitioning of ^3^H_2_O and the nonmetabolizable [^14^C]PEG, as in a previously described method (62).

### Transmembrane pH and membrane potential measurements.

Overnight cultures of *S. uberis* were diluted to an OD_600_ of 0.1 in THB (50 ml) at pH 7.5 and grown to an OD_600_ between 0.5 and 1.0. For ΔpH experiments, cells were harvested by centrifugation (3,220 × *g* for 20 min at 4°C), washed twice in THB (pH 5.0), and resuspended to an OD_600_ of 1.0. For ΔΨ experiments, cells were harvested under the same centrifugation conditions and then resuspended to an OD_600_ of 1.0 in THB (pH 7.5). In both experiments, cultures were then energized by incubating with glucose (20 mM) for 1 h at 37°C and 200 rpm. Aliquots of energized cells were either untreated or treated with membrane-permeabilizing toluene (0.4% [vol/vol]) or PBT2+Zn at 1× or 10× the CIC (0.5 mg/liter PBT2 + 10 μM zinc) for a further 20 min (37°C, 200 rpm). Following this, 1-ml aliquots were incubated in glass tubes with either [7-^14^C]benzoate (11 μM final concentration) or [^14^C]TPP^+^ (5 nM final concentration) for 5 min at 37°C. Radioisotope measurements and calculations of the ΔpH and ΔΨ were performed as previously described (62). Assays were undertaken in biological triplicates.

### Preparation of pyranine-containing liposomes.

Pyranine-containing liposomes were prepared according to the previously described method by Hards et al. (16). Liposomes were prepared in an incorporation buffer (5 mM MES [morpholineethanesulfonic acid]-MOPS [morpholinepropanesulfonic acid]-Tris in required proportions for desired pH) with or without zinc sulfate (1 mM).

### Quantification of internal pH by pyranine fluorescence.

The internal pH of liposomes was measured as previously described (16). A standard curve of the pyranine fluorescence ratio to pH was determined for each incorporation buffer, in the presence of 0.5 μM pyranine, at known pH values. Kinetic traces were measured on a Varioskan Flash plate reader (Thermo Scientific), and data were presented from the 15-min endpoint measurements. Carbonyl cyanide 3-chlorophenylhydrazone (CCCP; 1 μM) equilibrated the internal pH of liposome preparations with the external pH (Fig. S8), confirming the responsiveness of our preparations to pH gradients.

10.1128/mSphere.00157-20.8 FIG S8 Response of liposome preparations to a pH gradient. Pyranine-containing liposomes were prepared with an internal pH of 7.7 and suspended in MES-MOPS-Tris buffer at pH 7.7, 6.5, or 6.5 with carbonyl cyanide 3-chlorophenylhydrazone (CCCP) and measured for change in internal pH. Error bars represent the standard deviations from triplicate measurements. Download FIG S8, TIF file, 0.2 MB. Copyright © 2020 Harbison-Price et al. 2020 Harbison-Price et al. This content is distributed under the terms of the Creative Commons Attribution 4.0 International license.

### Isothermal titration calorimetry.

ITC (isothermal titration calorimetry) experiments were performed at 30°C with continuous stirring using a VP-ITC (GE Healthcare). ZnSO_4_·7H_2_O (zinc) and PBT2 were dissolved in 100 mM MOPS-Tris buffer (pH 7.7, 1% DMSO). The zinc sample (0.3 mM) was injected (30 × 10 μl) into PBT2 (0.035 mM), and the data were analyzed using Origin 7 software and fitted to a single site-binding model.

### RNA extraction and real-time PCR.

Biological triplicates of *S. uberis* NZ01 cells were grown to mid-log phase (OD_600_ of 0.3) in THB (45 ml) and treated for 1 h with either PBT2 (1.0 mg/liter), zinc (100 μM), PBT2 and zinc combined, or untreated. Total RNA was isolated using TRIzol-chloroform extraction as described previously (63) and purified using the RNA clean and concentrator-5 extraction kit (ZYMO research). Following elution of RNA into DNase/RNase-free H_2_O, RNA was DNase treated using the TURBO DNA-free kit (Invitrogen) according to the manufacturer’s instructions, and RNA was quantified on a NanoDrop instrument (Thermo Scientific).

cDNA for each sample was synthesized using a SuperScript III reverse transcriptase kit (Invitrogen). Primers used for quantitative PCR (qPCR) were designed on Primer-BLAST and are detailed in Table S2 in the supplemental material. Primer optimization and efficiency assays were performed, and qPCR experiments were conducted in a ViiA7 real-time PCR system (Applied Biosystems) using the PowerUp SYBR green master mix (Applied Biosystems) according to the manufacturer’s instructions. Results were normalized to the gene *pflC* using the threshold cycle (ΔΔ*C_T_*) method (64).

10.1128/mSphere.00157-20.10 TABLE S2 Real-time qPCR primers used in this study. Download Table S2, DOCX file, 0.1 MB. Copyright © 2020 Harbison-Price et al. 2020 Harbison-Price et al. This content is distributed under the terms of the Creative Commons Attribution 4.0 International license.

### SOD activity assays.

Overnight cultures were inoculated to an OD_600_ of 0.05 in THB and grown to an OD_600_ of 0.3. Cultures were split into 50-ml aliquots and treated for 1 h with PBT2 (1.0 mg/liter), zinc (100 μM), or manganese (800 μM), individually or in combination, or were untreated. Cultures were then harvested by centrifugation (3,220 × *g* for 10 min at 4°C), washed with HEPES buffer (50 mM, pH 7.4), resuspended in 1 ml of HEPES buffer, and lysed mechanically by bead beating three times (48,000 rpm, 30 s). Lysates were centrifuged (17,000 × *g*, 2 min, 4°C) and assayed for SOD activity using a SOD assay kit (Invitrogen) according to the manufacturer’s instructions. Protein concentrations in the lysates were determined by a BCA assay, and SOD activity was normalized for total protein content.

### H_2_O_2_ measurement.

H_2_O_2_ was measured using the Amplex Red hydrogen peroxide/peroxidase assay kit (Invitrogen) according to the manufacturer’s instructions. An overnight culture of *S. uberis* cells was diluted to an OD_600_ of 0.05 in THB and grown to mid-log phase (OD_600_ of 0.3). Mid-log-phase cells were separated into aliquots (10 ml) and were treated for 1 h with PBT2 (1.0 mg/liter), zinc (100 μM), or manganese (800 μM), individually or in combination, H_2_O_2_ (1 mM), or untreated. Cells were then harvested by centrifugation (3,220 × *g* for 7 min at 4°C). Cell pellets were washed twice in PBS under the same centrifugation conditions and resuspended in 1× reaction buffer at an OD_600_ of ∼3.5. For analysis, samples were diluted to an OD_600_ of ∼0.4 in 1× reaction buffer and Amplex Red reagent/horseradish peroxidase (HRP) working solution in a black-walled, clear-bottomed 96-well microplate. Amplex Red fluorescence was then measured using a Varioskan Flash microplate reader at excitation and emission wavelengths (λ_ex_ and λ_em_) of 560 and 590 nm, respectively.
