# Supplementary figures and images for: Multiple Bactericidal Mechanisms of the Zinc Ionophore PBT2
Source: mSphere. 2020 Mar 18;5(2):e00157-20. doi: 10.1128/mSphere.00157-20 (PMC7082140; doi:10.1128/mSphere.00157-20)

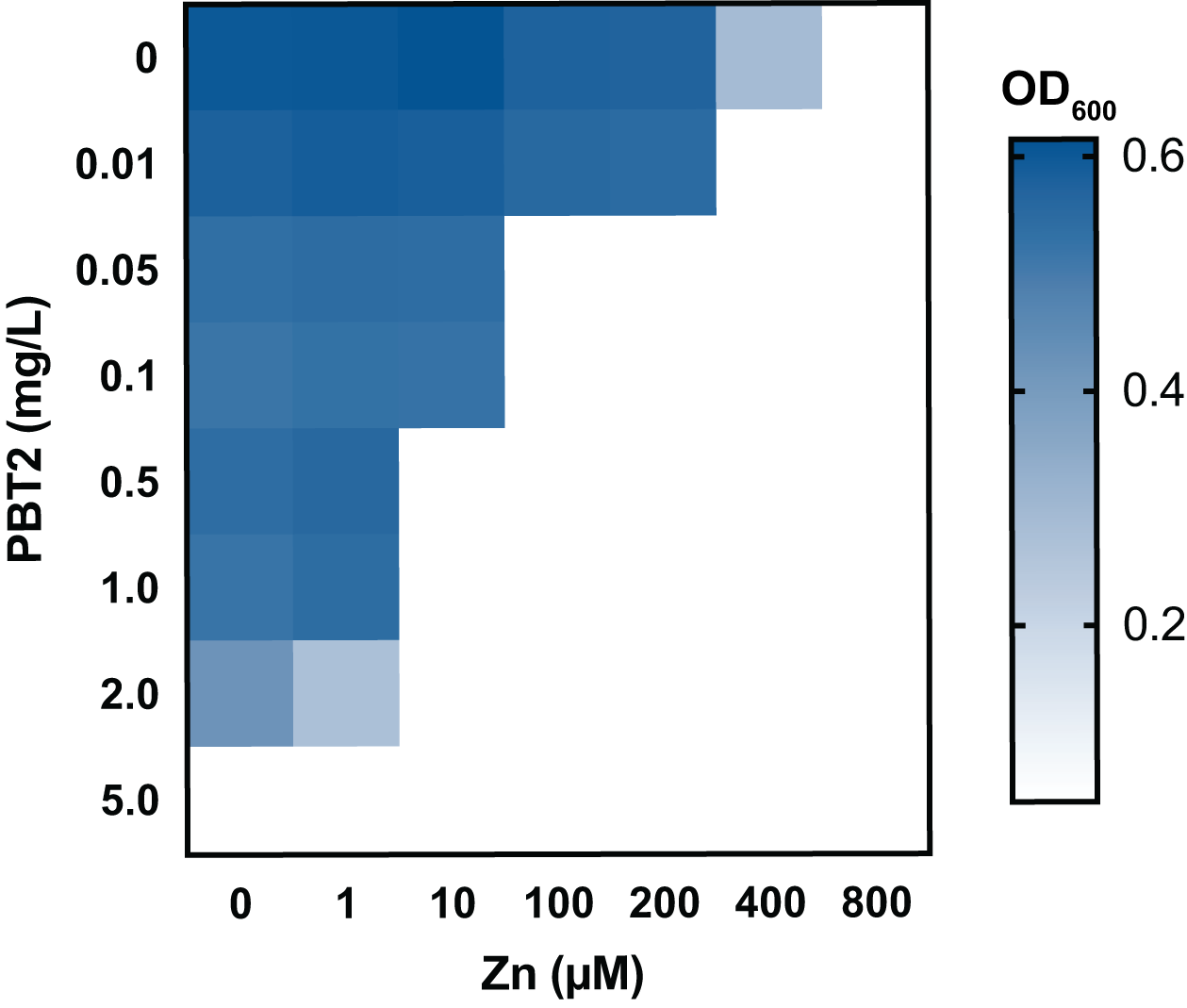

Supplement: FIG S1 [file mSphere.00157-20-sf001.tif]

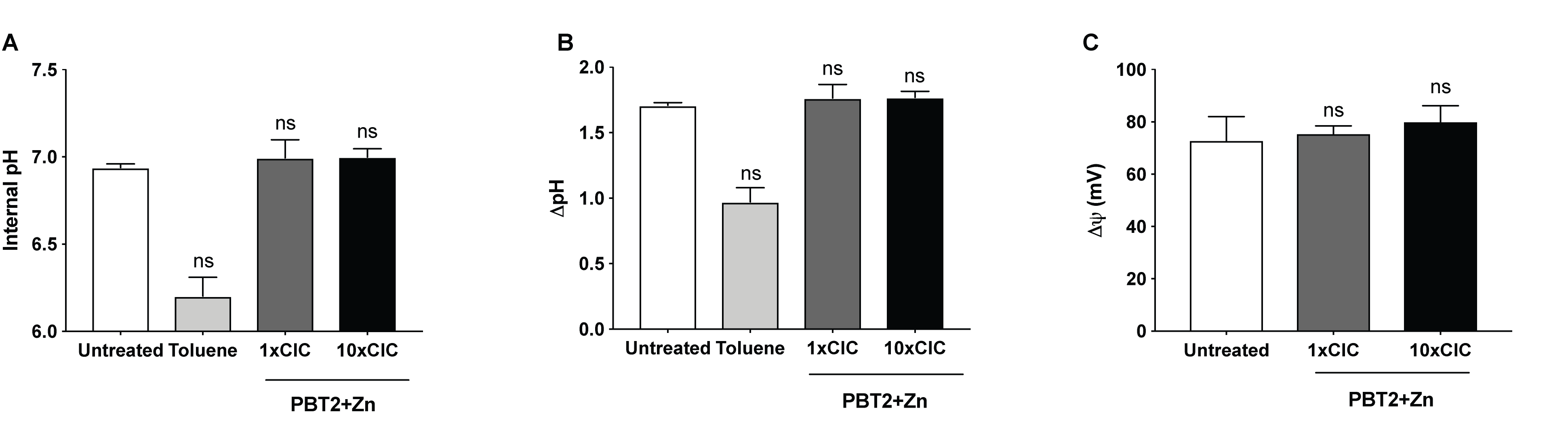

Supplement: FIG S2 [file mSphere.00157-20-sf002.tif]

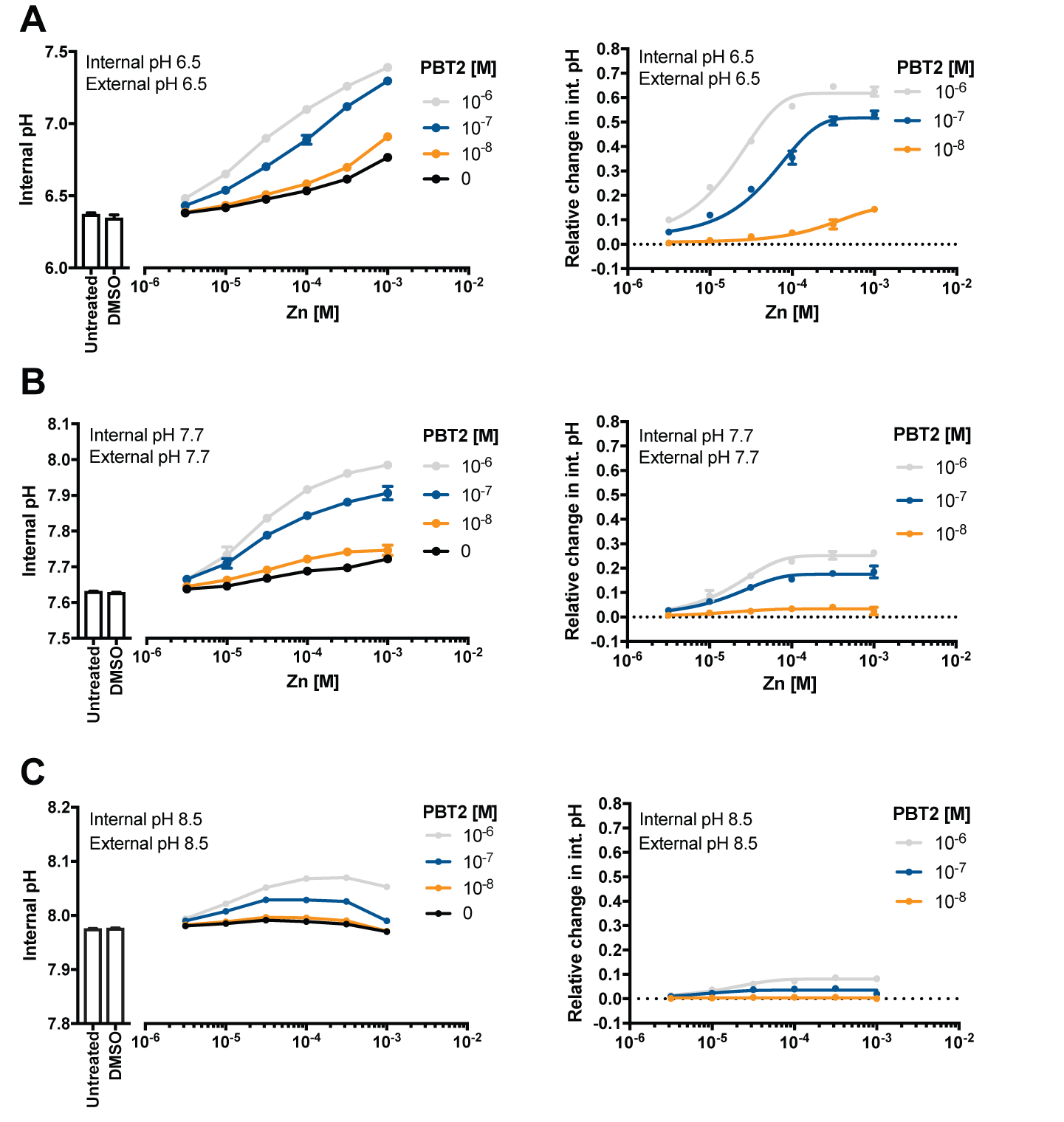

Supplement: FIG S3 [file mSphere.00157-20-sf003.tif]

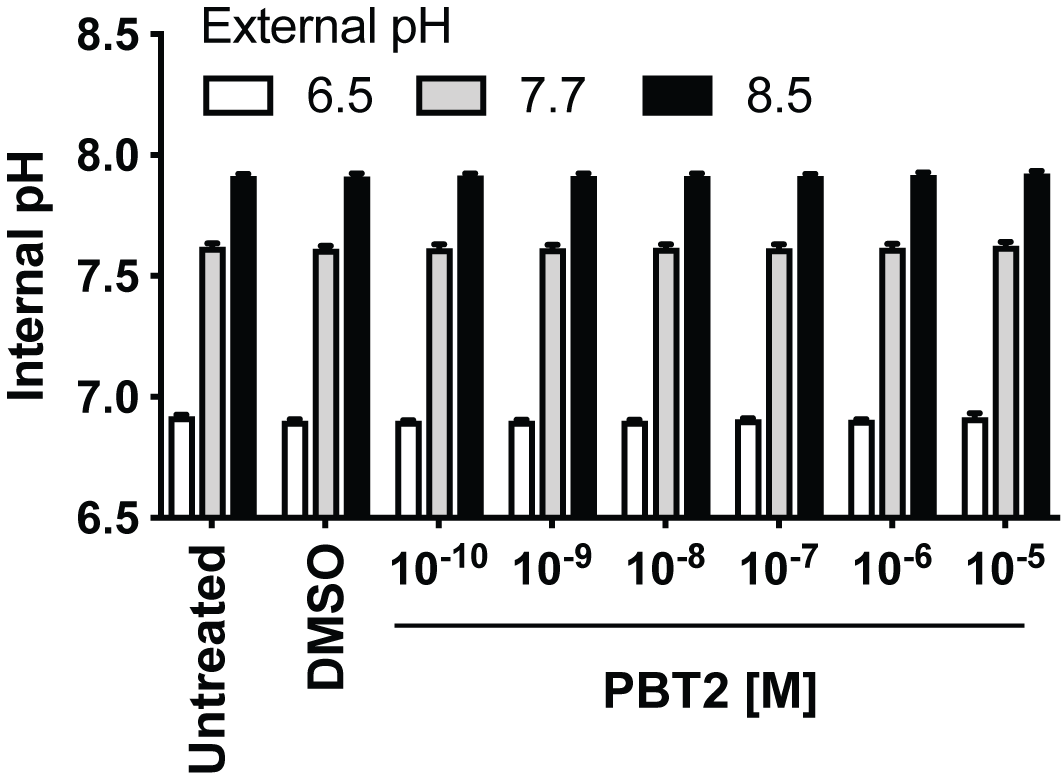

Supplement: FIG S4 [file mSphere.00157-20-sf004.tif]

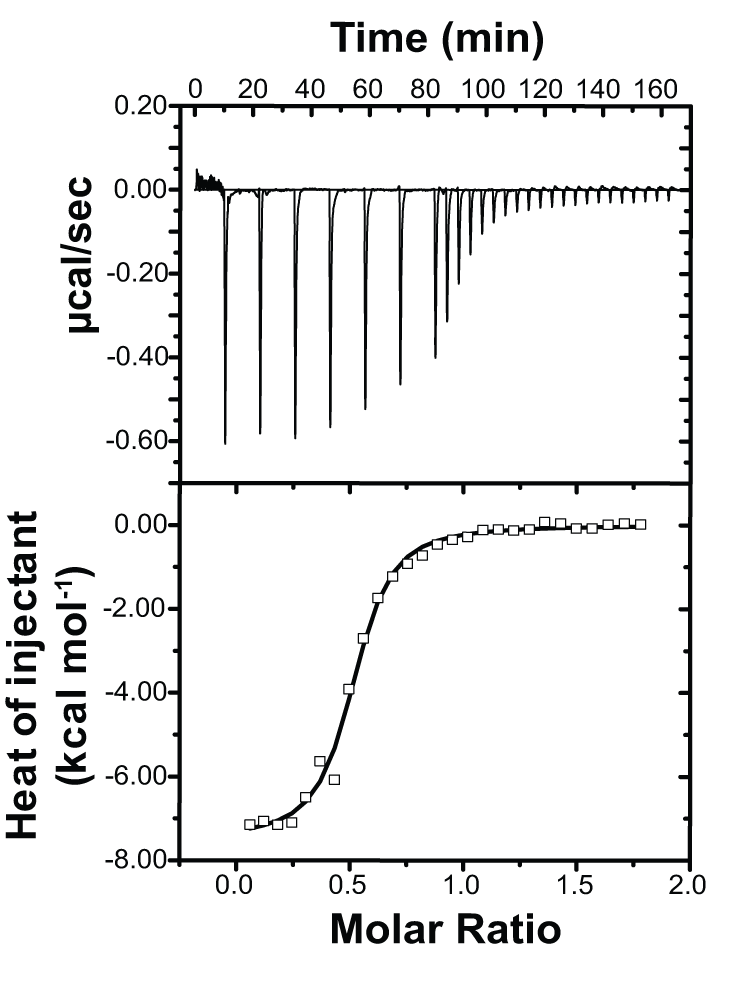

Supplement: FIG S5 [file mSphere.00157-20-sf005.tif]

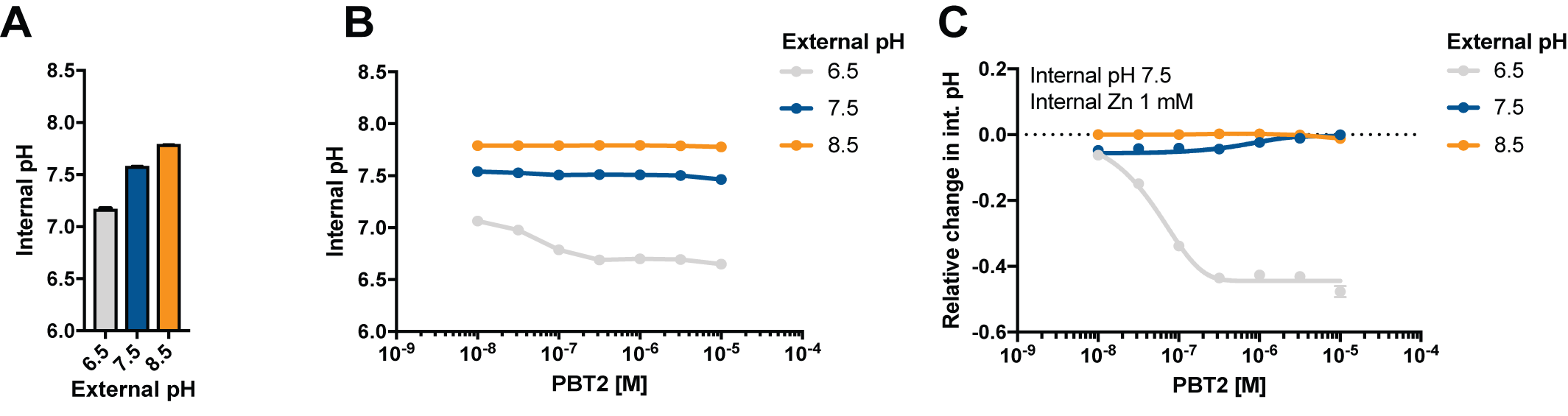

Supplement: FIG S6 [file mSphere.00157-20-sf006.tif]

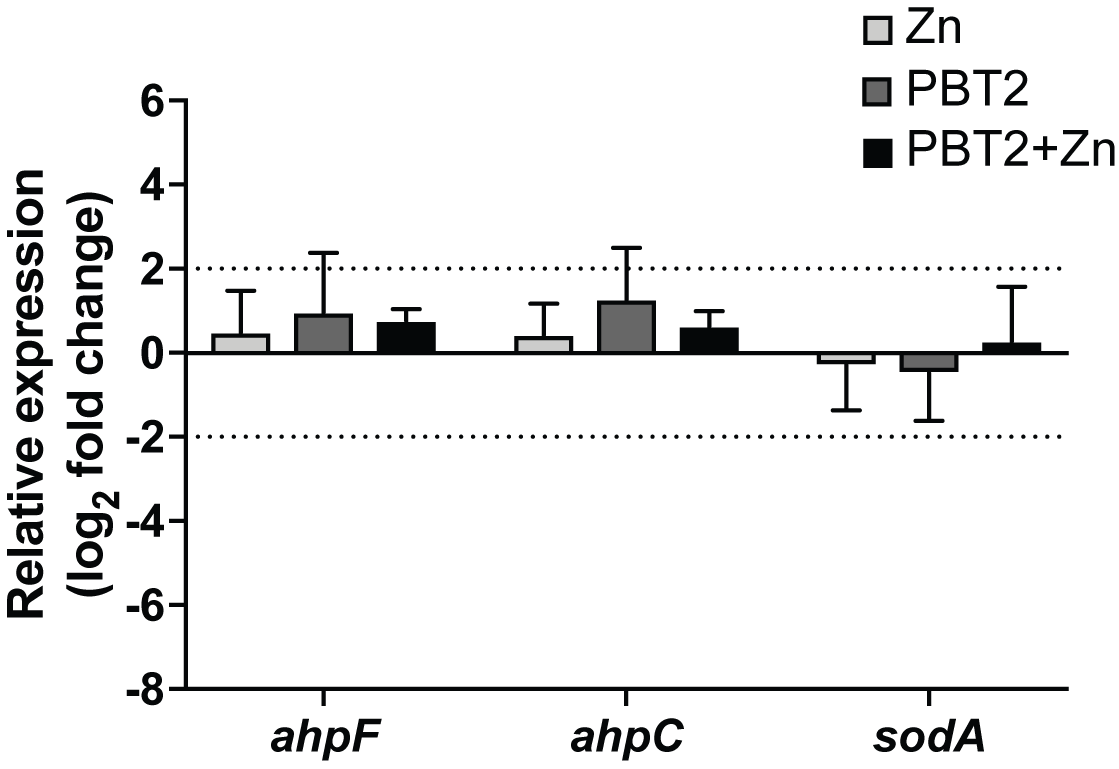

Supplement: FIG S7 [file mSphere.00157-20-sf007.tif]

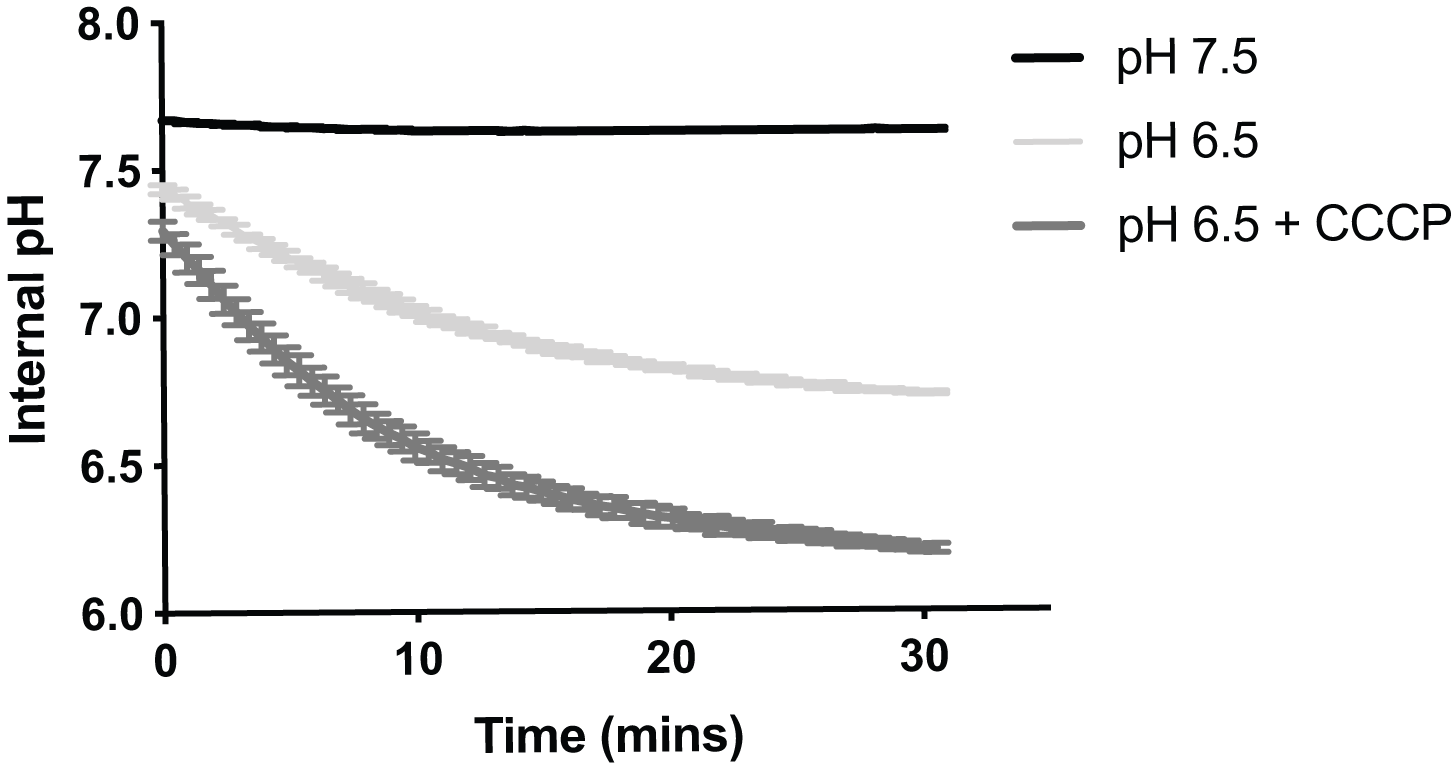

Supplement: FIG S8 [file mSphere.00157-20-sf008.tif]
