# Supplementary material for: Multiple Bactericidal Mechanisms of the Zinc Ionophore PBT2
Source: mSphere. 2020 Mar 18;5(2):e00157-20. doi: 10.1128/mSphere.00157-20 (PMC7082140; doi:10.1128/mSphere.00157-20)
Supplement: TABLE S1 [file mSphere.00157-20-st001.docx]

**Table S1. PBT2 and zinc exhibit antibacterial synergy against *S. uberis***

|  | **MIC of each compound** | | | |
| --- | --- | --- | --- | --- |
| **PBT2** | 5.0 mg/L | | | |
| **ZnSO_4_•7H_2_O** | 800 µM | | | |
|  | **CIC** | **FIC_PBT2_** | **FIC_Zn_** | **FICI** |
| **PBT2+ZnSO_4_•7H_2_O** | 0.05 mg/L PBT2  + 100 µM Zn | 0.01 | 0.125 | 0.135 |
|  | 0.5 mg/L PBT2  + 10 µM Zn | 0.1 | 0.0125 | 0.113 |

^†^ MIC, Minimum Inhibitory Concentration; CIC, Combined Inhibitory Concentration; FIC, Fractional Inhibitory Concentration; ^‡^ FICI, Fractional Inhibitory Concentration Index. ­
