# Supplementary material for: Multiple Bactericidal Mechanisms of the Zinc Ionophore PBT2
Source: mSphere. 2020 Mar 18;5(2):e00157-20. doi: 10.1128/mSphere.00157-20 (PMC7082140; doi:10.1128/mSphere.00157-20)
Supplement: TABLE S2 [file mSphere.00157-20-st002.docx]

**Table S2. Real-time qPCR primers used in this study**

| **Gene** | **Primer direction** | **Primer sequence (5’ → 3’)** | **Primer efficiency** |
| --- | --- | --- | --- |
| *mtuA* | Fwd | TGAACCGCTACCTGAAGATGT | 92.1% |
|  | Rev | TCAACACCATCACTGACAGCA |  |
| *fetB* | Fwd | TCAAATGATCCCTGTTGGTGGT | 101.8% |
|  | Rev | TATCAGCTCCCAAGGCCAAC |  |
| *cadA* | Fwd | TGTTCTGGGTTGGACTTGGT | 97.24% |
|  | Rev | CTTTCTGGCAGCTCGGCTAA |  |
| *adcA* | Fwd | GCAAATGAAGCGGGTGTCAA | 100.5% |
|  | Rev | TTACCTTCGCGTTCGGTTGT |  |
| *czcD* | Fwd | TCGATGCACACTTGGTGAGT | 110.7% |
|  | Rev | TCCAATCCATCTGCCGACAA |  |
| *sodA* | Fwd | TTGCTAATGCCAATGCTGCG | 96.1% |
|  | Rev | TGGTTAAGATGTCCGCCTCC |  |
| *ahpC* | Fwd | AAGCTTGGCATGATGACTCC | 90.9% |
|  | Rev | TGCTGACGAACGTATTGAGC |  |
| *ahpF* | Fwd | GTCTTTCATTCTGGCCGTGC | 108.7% |
|  | Rev | GCATAAATGGCTGCGCTGTT |  |
